# Demographic Characteristics, Perinatal Smoking Patterns, and Risk for Neonatal Health Complications Among Pregnant Smokers in the United States Who Begin Using Electronic Cigarettes During Pregnancy: A Descriptive Study Using Population-Based Surveillance Data

**DOI:** 10.1093/ntr/ntae119

**Published:** 2024-05-23

**Authors:** Hui Nian, Rachel Odland, Samantha Mindlin, Lin Ammar, Hilary Tindle, Angela M Miller, Kelli K Ryckman, Ethan Xie, Tina V Hartert, Brittney M Snyder, Steven M Brunwasser, Pingsheng Wu

**Affiliations:** Department of Biostatistics, Vanderbilt University Medical Center, Nashville, TN, USA; Osteopathic Medicine, Rowan-Virtua School of Osteopathic Medicine, Stratford, NJ, USA; Osteopathic Medicine, Rowan-Virtua School of Osteopathic Medicine, Stratford, NJ, USA; Epidemiology, Vanderbilt University School of Medicine, Nashville, TN, USA; Department of Medicine, Vanderbilt University Medical Center, Nashville, TN, USA; The Vanderbilt Center for Tobacco, Addiction and Lifestyle, Vanderbilt University Medical Center, Nashville, TN, USA; Geriatric Research Education and Clinical Centers, Veterans Affairs Tennessee Valley Healthcare System, Nashville, TN, USA; Division of Population Health Assessment, Tennessee Department of Health, Nashville, TN, USA; Department of Epidemiology and Biostatistics, Indiana University School of Public Health - Bloomington, Bloomington, IN, USA; Department of Epidemiology, University of Iowa, Iowa City, IA, USA; Department of Medicine, Vanderbilt University Medical Center, Nashville, TN, USA; Department of Molecular and Cell Biology, College of Letters and Science, University of California at Berkeley, Berkeley, CA, USA; Department of Medicine, Vanderbilt University Medical Center, Nashville, TN, USA; Department of Pediatrics, Vanderbilt University Medical Center, Nashville, TN, USA; Department of Medicine, Vanderbilt University Medical Center, Nashville, TN, USA; Department of Medicine, Vanderbilt University Medical Center, Nashville, TN, USA; Department of Psychology, Rowan University, Glassboro, NJ, USA; Department of Biostatistics, Vanderbilt University Medical Center, Nashville, TN, USA; Department of Medicine, Vanderbilt University Medical Center, Nashville, TN, USA

## Abstract

**Introduction:**

Health agencies have called for research evaluating e-cigarette (EC) use in supporting prenatal smoking cessation. This study aimed to describe (1) the characteristics of smokers who begin using electronic cigarettes (ECs) during pregnancy, (2) how frequently smokers reduce or eliminate pre- and post-natal combustible cigarette (CC) use, and (3) the risk for neonatal health complications among smokers who initiate ECs during pregnancy.

**Aims and Methods:**

Pregnant women using CCs exclusively during prepregnancy, who participated in a U.S. surveillance study, were classified by their reported late-pregnancy smoking behavior as CC-exclusive users, EC initiators, or quitters. EC initiators were further subclassified as dual users (used both ECs and CCs) or EC replacers (used ECs exclusively).

**Results:**

Of 29 505 pregnant smokers, 1.5% reported using ECs during the last three pregnancy months. Among them, 29.7% became EC-exclusive users. EC initiators were disproportionately non-Hispanic White. Relative to quitters, EC initiators had lower income, were less likely to be married, have intended pregnancies, receive first-trimester prenatal care, and participate in a federal assistance program. Compared to CC-exclusive users, EC initiators overall, and dual users specifically, were more likely to reduce pre- and post-natal CC usage relative to prepregnancy levels. EC initiators’ risk for neonatal health complications fell between quitters and CC-exclusive users, though the differences were not statistically significant.

**Conclusions:**

Although EC initiators reduced CC use more than CC-exclusive users, only 29.7% reported complete CC cessation, and there was insufficient evidence of reduction in neonatal health complications relative to CC-exclusive users. Currently, ECs should not be considered a viable gestational smoking cessation strategy.

**Implications:**

Health agencies have identified a critical need for research evaluating the use of e-cigarettes in supporting prenatal smoking cessation. Using the U.S. Pregnancy Risk Assessment Monitoring System surveillance study data, we provide real-world evidence that prenatal e-cigarette initiation as a smoking cessation tool is used infrequently among pregnant CCs smokers. Most using e-cigarettes in the last 3 months of pregnancy also used CCs.

## Introduction

Despite downward trends in cigarette use, tobacco remains the leading cause of preventable disease, disability, and death among pregnant individuals and their children in the United States and worldwide.^1^ Approximately 1 in 14 U.S. birthing individuals report prenatal smoking.^1^ Prenatal cigarette exposure is associated with increased risk for a host of offspring adverse health outcomes.^2–4^ Interventions supporting prenatal smoking abstinence could substantially reduce the public health burden.^1^ The U.S. Preventive Services Task Force (USPSTF) recognizes only behavioral approaches as established prenatal smoking cessation interventions.^5^

Pregnant smokers commonly perceive electronic cigarettes (ECs) to be less harmful than traditional combustible cigarettes^6,7^ (CCs) and a viable CC cessation or reduction tool.^6,8^ The 2022 Cochrane review concluded that nicotine-containing ECs are effective for smoking cessation.^9^ However, most studies included were conducted in the general population, and the safety and efficacy of ECs as a smoking cessation tool compared to behavioral approaches in pregnancy are poorly established. Little is known about whether prenatal EC initiation promotes CC abstinence, and in fact, there is accumulating evidence linking prenatal EC use to adverse neonatal health outcomes.^10–14^ The USPSTF in 2021 called for research evaluating whether ECs are a viable smoking cessation tool and to uncover patterns and potential dangers of e-cigarette use in pregnancy.^5^ Consequently, we aimed to close this important research gap by addressing the following questions:

1. What proportion of CC-exclusive smokers use ECs during late pregnancy (last 3 months)?
2. What proportion of EC initiators quit CCs altogether versus becoming dual CC/EC users?
3. Do EC initiators reduce the amount of prenatal and post-natal CC use more than late-pregnancy CC-exclusive users?
4. What demographic and health characteristics are associated with late-pregnancy EC use?
5. How common are adverse perinatal outcomes among EC initiators relative to those who quit smoking and those who smoke CCs exclusively during late pregnancy?

## Materials and Methods

### Data Source and Study Population

We studied pregnant individuals with singleton live births between 2016 and 2020 who participated in the U.S. Centers for Disease Control and Prevention’s (CDC) Pregnancy Risk Assessment Monitoring System (PRAMS) survey, an ongoing surveillance study of individuals with recent live births. Participants complete the PRAMS survey 2–6 months post-delivery, providing population-based information on maternal attitudes and experiences before, during, and shortly after pregnancy. The data includes demographic and medical information ascertained through linked birth certificates. Detailed methods regarding the PRAMS study design have been described elsewhere.^15^

PRAMS captured the daily average number of CCs consumed in the 3 months before pregnancy, the last 3 months of pregnancy, and the 2–6 months post-delivery (Table S1). ECs (e-cigarettes and other electronic nicotine products such as vape pens, e-hookahs, hookah pens, e-cigars, and e-pipes) are defined in the PRAMS survey as battery-powered devices that use nicotine liquid rather than tobacco leaves and produce vapor instead of smoke. Frequency of EC use in the 3 months before pregnancy and the last 3 months of pregnancy was captured (Table S1). Our study included a subsample of 29, 505 PRAMS participants (representing a weighted sample of 1 380 939) who self-reported CC use without EC use in the 3 months before pregnancy. As our goal was to inform decisions about whether prenatal EC initiation is a reasonable strategy for reducing harmful prenatal CC exposure, we excluded women who reported EC use (either mono-EC use or dual-EC/CC use) 3 months prior to pregnancy. Our study included 5 years of data (2016–2020) from 48 sites meeting the PRAMS’ minimum overall response rate threshold policy. The CDC PRAMS Working Group and the Vanderbilt University Institutional Review Board (#191880) approved the study.

### Statistical Analysis

PRAMS implements site-specific stratified random sampling and provides analysis weights accounting for complex sampling design, nonresponse patterns, and noncoverage rates.^15^ Analyses were conducted using the R software^16^ package “survey”^17^ to incorporate analysis weights.

We classified pregnant smokers into one of three mutually exclusive groups based on their self-reported late pregnancy (last 3 prenatal months) smoking behavior. *CC-exclusive users:* reported exclusive CC use; *EC initiators:* Reported using ECs. *Quitters:* Reported neither EC nor CC use; EC initiators were further classified into two mutually exclusive subgroups: *Dual users:* used CCs in addition to ECs; *EC replacers*: Discontinued CC use and only used ECs (Figure S1).

We performed descriptive analysis evaluating differences among the groups in demographics, health behaviors, preexisting health conditions, perinatal outcomes, and average daily CC use before, during, and after pregnancy. Change in the average daily CC use from before pregnancy to late pregnancy and after pregnancy were described. Unweighted frequencies and weighted population percentage estimates and their 95% confidence intervals (CI) were reported. Relative standard errors—a measure of reliability—of the weighted population percentage estimates were calculated as the percentage of the standard errors of the point estimates.^18^ Comparisons between CC-exclusive users, quitters, and EC initiators were conducted using the Rao-Scott Chi-square test (α = 0.05). If the overall test was significant, post hoc pairwise comparisons were conducted with Holm adjustment to the *p*-values. Cell sizes in the two EC-initiators subgroups were generally smaller than the PRAMS’ guideline for statistical comparisons (*n* = 50), so we reported characteristics for these groups without statistical comparisons. We performed survey-weighted multivariable logistic regressions with inverse-probability weighting and design-based standard errors to assess the odds of having a preterm birth, low birth weight, and small-for-gestational-age (SGA) adjusting for plausible confounders: maternal age, race/ethnicity, education level, household income, marital status, prenatal participation in the WIC program, pregnancy intention, flu vaccine receipt, the Kotelchuck index, initiation of prenatal care in the first trimester, parity, history of preterm birth, maternal prepregnancy BMI, preexisting and/or gestational hypertension, preexisting and/or gestational diabetes, self-reported diagnosis of depression before and/or during pregnancy, and year of delivery. The odds of these adverse outcomes for the EC initiators were compared to those of the CC-exclusive users and quitters. Participants were dropped from specific analyses involving variables for which they had missing data, but analyses took into consideration the corresponding weights for individuals with missing covariates.^17^

We evaluated differences across prenatal smoking groups in demographic characteristics: Age at delivery, race/ethnicity, education level, household income, and marital status. Participant care and health behavior characteristics: Pregnancy intention, prenatal participation in the Special Supplemental Nutrition Program for Women, Infants, and Children (WIC), flu vaccine receipt prior to delivery, the Kotelchuck index^19^ of prenatal care adequacy, commencement of prenatal care in the first trimester, and breastfeeding status; and participant health conditions and related characteristics: Maternal prepregnancy body mass index (BMI), parity, history of preterm birth, preexisting and/or gestational hypertension, preexisting and/or gestational diabetes, and self-reported diagnosis of depression before and/or during pregnancy. We further included pregnancy outcomes of delivery method and plurality, and adverse neonatal outcomes of preterm birth, low birth weight, and SGA.

## Results

Overall, 68.1% of the weighted population (*N* = 1 380 939) identified as Non-Hispanic White, 15.1% as Non-Hispanic Black, 7.1% as Hispanic, and 9.7% as Other. Most participants at delivery were in the 25–34 years old age group (57.5%). Over half of the sample did not attend college (56.7%; Table 1, Figure S2).

**Table 1.** Participant Demographic Characteristics

Characteristics	CC-exclusive user			EC initiator			Quitter			*p*-value
	*N* ^a^	% (95% CI)^b^	RSE (%)^c^	*N* ^a^	% (95% CI)^b^	RSE (%)	*N* ^a^	% (95% CI)^b^	RSE (%)	
Maternal age	14 095			395			15 013			.367
<=24	3785	28.3 (27.0, 29.6)	2.4	113	31.6 (23.8, 40.2)	12.6	4512	29.9 (28.6, 31.1)	2.1	
25–34	8248	58.6 (57.2, 60.0)	1.2	229	54.7 (46.2, 63.1)	7.6	8403	56.7 (55.4, 58.0)	1.2	
≥35	2062	13.1 (12.1, 14.1)	3.7	53	13.7 (8.8, 20.0)	19.8	2098	13.4 (12.6, 14.3)	3.2	
Maternal race/ethnicity	14 005			392			14 908			<.001
Non-Hispanic White	7650	71.8 (70.6, 73.0)	0.9	301	89.1 (84.3, 92.8)	2.3	7213	64.4 (63.1, 65.6)	1.0	
Non-Hispanic Black	2660	15.1 (14.1, 16.1)	3.3	22	2.5 (1.2, 4.7)	32.3	2714	15.4 (14.5, 16.3)	3.1	
Hispanic	681	4.6 (4.0, 5.2)	6.4	14	2.6 (0.8, 6.0)	44.9	1420	9.4 (8.6, 10.2)	4.2	
Other	3014	8.4 (7.8, 9.2)	4.2	55	5.8 (3.2, 9.6)	26.3	3561	10.9 (10.1, 11.6)	3.6	
Maternal education	13 941			394			14 915			<.001
Some high school or less	3404	23.6 (22.4, 24.9)	2.7	63	15.6 (10.4, 22.2)	18.3	2041	12.5 (11.6, 13.5)	3.7	
High school graduate	5840	41.8 (40.4, 43.3)	1.7	151	38.7 (30.6, 47.2)	10.5	5081	34.5 (33.2, 35.8)	1.9	
Some college or more	4697	34.6 (33.2, 35.9)	2.0	180	45.7 (37.4, 54.2)	9.1	7793	53.0 (51.6, 54.3)	1.3	
Household income	12 987			372			13 950			<.001
<$20 000	7984	58.2 (56.7, 59.6)	1.3	214	52.4 (43.6, 61.1)	8.2	5701	35.8 (34.5, 37.2)	1.9	
$20 000–$40 000	3038	24.9 (23.6, 26.2)	2.6	87	26.4 (18.7, 35.2)	15.2	3495	25.1 (23.8, 26.3)	2.5	
> 40 000	1965	16.9 (15.8, 18.1)	3.3	71	21.2 (14.8, 28.9)	16.2	4754	39.1 (37.8, 40.4)	1.7	
Marital status	14 054			395			15 001			<.001
Married	3722	28.0 (26.7, 29.3)	2.3	108	28.0 (21.0, 35.9)	13.0	5677	41.4 (40.1, 42.7)	1.6	
Other	10 332	72.0 (70.7, 73.3)	0.9	287	72.0 (64.1, 79.0)	5.1	9324	58.6 (57.3, 59.9)	1.1	
Year of delivery	14 096			395			15 014			.174
2016	2572	19.8 (18.7, 21.0)	3.0	76	17.9 (12.1, 25.1)	17.6	2869	20.6 (19.6, 21.7)	2.7	
2017	2911	20.6 (19.5, 21.7)	2.7	72	15.4 (10.6, 21.2)	16.9	3230	21.0 (20.0, 22.0)	2.5	
2018	3191	22.3 (21.2, 23.5)	2.6	83	24.8 (17.9, 32.9)	14.7	3352	21.7 (20.6, 22.7)	2.4	
2019	2853	19.9 (18.8, 21.1)	2.9	84	21.7 (15.2, 29.5)	16.0	3066	20.9 (19.8, 22.0)	2.7	
2020	2569	17.4 (16.3, 18.4)	3.1	80	20.3 (13.8, 28.1)	17.1	2497	15.8 (14.9, 16.8)	3.0	

^a^Unweighted sample size.

^b^Weighted prevalence and 95% confidence interval.

^c^Relative standard error of weighted prevalence with higher value indicates statistically unreliable estimation. Statistical analysis by a Rao-Scott Chi-square test.

CC = combustible cigarette; EC = e-cigarette; RSE = relative standard error.

### Smoking Behavior During and After Pregnancy

Approximately half (53.9%) of the sample stopped smoking cigarettes altogether during pregnancy (quitters). Forty-five percent used CCs exclusively in late pregnancy (CC-exclusive users), whereas only 1.5% used ECs in late pregnancy (EC initiators). Quitters smoked fewer CCs daily prior to pregnancy than the other smoking groups, whereas EC initiators smoked the most before pregnancy (Table 2, Figure S3). Most quitters (82.9%) reported smoking ≤ 10 CCs per day prior to pregnancy, compared to just over half of CC-exclusive users (52.8%) and a minority of EC initiators (36.6%). Of the EC initiators (1.5%), 70.3% were late-pregnancy dual users, and 29.7% discontinued CC use (EC replacers).

**Table 2.** Average Daily Use of Combustible Cigarettes (CCs) Before, During, and After Pregnancy

Average daily use	CC-exclusive user			EC initiator			Quitter			*p*-value
	*N* ^a^	% (95% CI)^b^	RSE (%)^c^	*N*	% (95% CI)	RSE (%)	*N*	% (95% CI)	RSE (%)	
Before pregnancy	14 096			395			15 014			<.001
0 cigarette	—	—		—	—		—	—		
1–<10 cigarettes	7878	52.8 (51.3, 54.2)	1.4	158	36.6 (28.7, 45.0)	10.9	12 670	82.9 (81.9, 83.9)	0.6	
10–20 cigarettes	4646	35.5 (34.2, 36.9)	2.0	170	45.4 (37.1, 53.9)	9.1	1871	13.9 (13.0, 14.9)	3.4	
> 20 cigarettes	1572	11.7 (10.7, 12.7)	4.1	67	18.0 (12.1, 25.1)	17.5	473	3.2 (2.7, 3.7)	7.8	
During pregnancy	14 096			395			15 014			<.001
0 cigarette	0	0	—	117	29.7 (22.6, 37.6)		15 014	100	—	
1–<10 cigarettes	12 022	85.0 (84.0, 86.0)	0.6	225	57.2 (48.9, 65.3)	7.1	0	0	—	
10–20 cigarettes	1657	12.2 (11.3, 13.2)	3.9	40	10.0 (5.9, 15.6)	22.9	0	0	—	
> 20 cigarettes	417	2.8 (2.3, 3.3)	8.8	13	3.1 (1.1, 6.8)	41.1	0	0	—	
After pregnancy	14 016			394			14 950			<.001
0 cigarette	1193	8.5 (7.7, 9.3)	4.7	108	30.1 (22.6, 38.6)	13.0	8734	60.0 (58.7, 61.3)	1.1	
1–<10 cigarettes	8500	57.2 (55.8, 58.7)	1.3	173	42.1 (34.0, 50.6)	9.7	5722	36.8 (35.5, 38.1)	1.8	
10–20 cigarettes	3538	28.4 (27.1, 29.7)	2.4	87	21.6 (15.3, 29.1)	15.6	421	2.8 (2.4, 3.2)	8.0	
> 20 cigarettes	785	5.9 (5.3, 6.6)	5.8	26	6.1 (2.9, 11.0)	30.8	73	0.4 (0.3, 0.6)	18.8	

^a^Unweighted sample size

^b^Weighted prevalence and 95% confidence interval.

^c^Relative standard error of weighted prevalence with higher value indicates statistically unreliable estimation. Statistical analysis by a Rao-Scott Chi-square test.

CC = combustible cigarette; EC = e-cigarette; RSE = relative standard error.

EC initiators were more likely than CC-exclusive users to report reduced late-pregnancy and post-natal CC use. Aside from the 29.7% of EC replacers who quit CCs during pregnancy, another 55.1% reported reduced CC use. After delivery, 61.1% of EC initiators either reported maintained CC abstinence or reduced CC usage relative to before pregnancy compared to 36.9% of CC-exclusive users (Figure S4). When excluding EC replacers, EC initiators who reported dual use were still more likely than CC-exclusive users to report reduced daily CC use in late pregnancy (dual user: 78.3% vs. CC-exclusive user: 62.1%) and after pregnancy (dual user: 54.3% vs. CC-exclusive user: 36.9%) relative to prepregnancy levels (Table S2, Figures S4 and S5).

### Maternal Demographics Characteristics

The percentage of non-Hispanic White participants was greater among EC initiators (89.1%) than CC-exclusive users (71.8%) or quitters (64.4%; Table 1). Compared to quitters, both EC initiators and CC-exclusive users were less likely to be married and had lower household incomes. CC-exclusive users had less education than both EC initiators and quitters (Figure S2).

### Maternal Health Behaviors and Care Characteristics

Regarding health behaviors (Table 3, Figure S6), both EC initiators and CC-exclusive users were less likely than quitters to report having intended pregnancies, receiving federal food and nutritional assistance, and initiating prenatal care during the first trimester. CC-exclusive users were less likely than quitters to report receiving a flu vaccine and having at least “adequate” prenatal care and less likely than both quitters and EC initiators to report breastfeeding.

**Table 3.** Participant Health Behavior and Care Characteristics and Health-Related Characteristics

Characteristics	CC-exclusive user			EC initiator			Quitter			*p*-value
	*N* ^a^	% (95% CI)^b^	RSE (%)^c^	*N* ^a^	% (95% CI)^b^	RSE (%)	*N* ^a^	% (95% CI)^b^	RSE (%)	
Pregnancy intention	13 856			386			14 775			<.001
Intended	4854	34.7 (33.3, 36.0)	2.0	139	35.4 (27.5, 43.8)	11.3	6788	47.7 (46.4, 49.1)	1.4	
Unintended	4757	36.2 (34.8, 37.7)	2.0	136	31.7 (24.1, 40.1)	12.3	4592	31.8 (30.6, 33.1)	2.0	
Not sure	4245	29.1 (27.8, 30.4)	2.3	111	32.9 (25.3, 41.3)	11.9	3395	20.5 (19.4, 21.6)	2.7	
WIC program participation	13 856			390			14 810			<.001
Yes	5377	40.0 (38.5, 41.4)	1.8	169	42.3 (34.2, 50.8)	9.6	7571	55.1 (53.8, 56.5)	1.2	
Flu vaccine receipt	13 851			391			14 792			<.001
Yes	6850	46.7 (45.2, 48.1)	1.6	195	47.8 (39.3, 56.3)	8.7	8331	53.6 (52.3, 55.0)	1.3	
Kotelchuck index	13 676			388			14 606			<.001
Inadequate	3150	21.2 (20.1, 22.5)	2.9	76	22.5 (15.4, 31.1)	16.9	2106	12.6 (11.7, 13.5)	3.6	
Intermediate	1587	11.0 (10.1, 11.9)	4.1	34	7.7 (4.5, 12.1)	23.7	1566	10.4 (9.6, 11.2)	4.1	
Adequate	4478	37.0 (35.6, 38.5)	1.9	140	37.6 (29.6, 46.1)	10.8	5752	43.1 (41.8, 44.5)	1.6	
Adequate plus	4461	30.7 (29.4, 32.1)	2.2	138	32.2 (24.7, 40.4)	11.9	5182	33.9 (32.6, 35.2)	1.9	
Prenatal care starts in the first trimester of pregnancy	13 742			387			14 783			<.001
Yes	10 442	78.5 (77.3, 79.7)	0.8	297	75.5 (67.3, 82.6)	4.9	12 682	87.2 (86.2, 88.1)	0.5	<.001
Breastfeeding	13 084			375			14 629			
Ever	9116	69.4 (68.0, 70.7)	1.0	304	82.3 (75.0, 88.1)	3.9	12 297	84.0 (83.0, 85.0)	0.6	
Prepregnancy BMI	13 642			387			14 709			<.001
Underweight	674	4.7 (4.2, 5.4)	6.4	21	4.3 (1.3, 10.2)	46.1	438	3.0 (2.5, 3.5)	8.0	
Normal	4996	36.0 (34.6, 37.4)	2.0	174	47.9 (39.5, 56.4)	8.7	5061	36.4 (35.1, 37.7)	1.8	
Overweight	3320	24.8 (23.5, 26.1)	2.6	98	25.5 (18.8, 33.2)	13.7	3807	26.7 (25.5, 27.9)	2.3	
Obese	4652	34.5 (33.1, 35.9)	2.0	94	22.3 (16.1, 29.6)	14.7	5403	34.0 (32.7, 35.2)	1.9	
Parity	14 067			394			14 989			<.001
Primiparous	3627	25.5 (24.3, 26.8)	2.4	132	34.8 (26.9, 43.5)	11.7	6146	42.2 (40.9, 43.5)	1.6	
2	3958	31.1 (29.7, 32.4)	2.2	120	29.9 (22.9, 37.6)	12.1	4414	31.9 (30.6, 33.1)	2.0	
≥3	6482	43.4 (42.0, 44.8)	1.7	142	35.3 (27.4, 43.8)	11.4	4429	25.9 (24.8, 27.1)	2.3	
History of preterm birth	14 056			392			14 984			<.001
Yes	1284	7.0 (6.3, 7.7)	5.1	23	3.6 (1.7, 6.7)	31.9	808	3.6 (3.2, 4.1)	6.1	
Maternal hypertension (preexisting and gestational)	13 899			390			14 838			.434
Yes	3240	20.4 (19.3, 21.5)	2.8	80	17.6 (12.0, 24.6)	17.3	3411	19.6 (18.6, 20.6)	2.6	
Maternal diabetes (type I/II/gestational)	13 850			389			14 796			.921
Yes	1890	13.2 (12.2, 14.3)	3.9	53	12.9 (8.1, 19.1)	20.5	2066	13.0 (12.1, 13.8)	3.4	
Maternal depression	13 863			391			14 771			<.001
Ever	5959	43.0 (41.6, 44.5)	1.7	206	55.2 (46.7, 63.4)	7.4	4517	28.4 (27.2, 29.7)	2.2	

^a^Unweighted sample siz.

^b^Weighted prevalence and 95% confidence interval.

^c^Relative standard error of weighted prevalence with higher value indicates statistically unreliable estimation. Statistical analysis by a Rao-Scott Chi-square test.

CC = combustible cigarette; EC = e-cigarette; RSE = relative standard error.

### Maternal Health-Related Characteristics

On indicators of participant health conditions (Table 3, Figure S7), EC initiators were less likely than both CC-exclusive users and quitters to be obese. EC initiators were less likely to be primiparous compared to quitters, followed by CC-exclusive users. Both EC initiators and quitters were more likely to have a history of preterm birth compared to CC-exclusive users. EC initiators were also more likely than both CC-exclusive users and quitters to report having a history of depression. The proportion of participants reporting a history of hypertension and diabetes did not differ significantly across the three groups.

### Delivery Related Outcomes and Neonatal Health Outcomes

There were several differences among the groups in the prevalence of neonatal health complications (Table 4, Figure S8). CC-exclusive users were at elevated risk for preterm birth (13.0%), low birth weight (13.3%), and SGA (19.3%) relative to quitters (preterm birth: 9.6%; low birth weight: 7.8%; SGA: 9.6%). EC initiators fell between quitters and CC-exclusive users in their level of risk for neonatal health complications (preterm birth: 10.6%; low birth weight: 10.6%, and SGA: 16.9%), though they did not differ significantly from the other groups once correcting for multiple comparisons in either unadjusted or covariate-adjusted analyses (Table S3). There were no group differences in the prevalence of c-sections or plurality (Figure S9).

**Table 4.** Perinatal and Neonatal Health Outcomes

Characteristics	CC-exclusive user			EC initiator			Quitter			*p*-value
	*N* ^a^	% (95% CI)^b^	RSE (%)^c^	*N*	% (95% CI)	RSE (%)	*N*	% (95% CI)	RSE (%)	
Delivery method	14 083			394			15 011			.088
Vaginal	9057	66.7 (65.3, 68.0)	1.0	251	65.1 (56.7, 72.8)	6.1	9488	64.6 (63.3, 65.9)	1.0	
C-section	5026	33.3 (32.0, 34.7)	2.0	143	34.9 (27.2, 43.3)	11.3	5523	35.4 (34.1, 36.7)	1.8	
Plurality	13 703			372			14 707			.681
Singleton	13 235	98.2 (97.9, 98.5)	0.2	358	96.8 (91.7, 99.2)	1.7	14 160	98.2 (97.9, 98.4)	0.2	
More than one	468	1.8 (1.5, 2.1)	8.4	14	3.2 (0.8, 8.3)	51.0	547	1.8 (1.6, 2.1)	7.9	
Preterm birth	14 051			394			14 997			<.001
No	10 710	87.0 (86.2, 87.8)	0.5	301	89.4 (84.6, 93.1)	2.3	12 238	90.4 (89.7, 91.1)	0.4	
Yes	3341	13.0 (12.2, 13.8)	3.2	93	10.6 (6.9, 15.4)	19.5	2759	9.6 (8.9, 10.3)	3.5	
Low birth weight	14 081			395			15002			<.001
No	9420	86.7 (86.0, 87.4)	0.4	270	89.4 (85.2, 92.7)	2.0	11 862	92.2 (91.7, 92.6)	0.3	
Yes	4661	13.3 (12.6, 14.0)	2.6	125	10.6 (7.3, 14.8)	17.1	3140	7.8 (7.4, 8.3)	3.1	
SGA	13 546			379			14 412			<.001
No	10 088	80.7 (79.6, 81.8)	0.7	293	83.1 (75.8, 89.0)	3.8	12 407	90.4 (89.6, 91.1)	0.4	
Yes	3458	19.3 (18.2, 20.4)	2.9	86	16.9 (11.0, 24.2)	18.9	2005	9.6 (8.9, 10.4)	3.9	

^a^Unweighted sample size

^b^Weighted prevalence and 95% confidence interval

^c^Relative standard error of weighted prevalence with higher value indicates statistically unreliable estimation. Statistical analysis by a Rao-Scott Chi-square test.

CC = combustible cigarette; EC = e-cigarette; RSE = relative standard error.

### Dual Users and EC Replacers

For comparisons of dual users versus EC replacers, nearly all variables had cells with < 50 participants. The small sample size often resulted in large relative standard errors, an indication of large sampling errors or low precision in the estimates, which precluded statistical comparisons (Tables S2, S4–S7). However, a notably smaller percentage of EC replacers had babies who were SGA (5.7%) relative to dual users (21.3%; Table S4, Figure S10).

## Discussion

### Main Findings

These findings could inform public health messaging and policy related to the use of ECs in pregnancy. First, despite the common perception among pregnant individuals that ECs are a safer alternative to CCs,^6,7^ only a small proportion of pregnant smokers (approximately 1.5%) reported using ECs during late pregnancy. Between 2016 and 2020, EC use in the last three gestational months in the United States (whether as a smoking cessation/reduction strategy or otherwise) was rare among those who smoked CCs exclusively for prepregnancy. For now, limited late-pregnancy EC use may allay concerns about the potential for widescale EC-related teratogenesis, though it will be important to confirm low usage rates earlier in pregnancy. Compared to CC-exclusive users and quitters, EC initiators used CCs the most prior to pregnancy. Second, approximately 70% of pregnant smokers who were using ECs in late pregnancy were dual users, rather than quitting CCs altogether. Third, EC initiators, and even dual users, were more likely than CC-exclusive users to report reduced daily CC use during pregnancy and after delivery relative to prepregnancy levels. Fourth, several notable demographic, behavioral, and health characteristics were correlated with EC initiation during pregnancy, which may reflect group differences in EC acceptance, perception of EC benefits, or EC access. Finally, whereas CC-exclusive users had elevated prevalence of preterm birth, low birth weight, and SGA relative to quitters, prevalence rates among EC initiators consistently fell between the two other smoking groups and were not significantly different in covariate-adjusted analyses correcting for multiple comparisons. There was considerable uncertainty in these comparisons as the EC-initiator group was by far the smallest.

Our findings provide real-world evidence and critically extend what is known about EC use in pregnancy. A prior UK study found that most pregnant smokers who used ECs continued to use CCs.^20^ Similarly, a smaller U.S. national sample found that EC use in early pregnancy did not predict subsequent CC abstinence.^21^ Finally, a prior PRAMS study found that EC users were more likely than their non-EC-using peers to use CCs and smoked at best a comparable number of cigarettes compared to CC-exclusive users.^10^ Thus, there is converging real-world evidence that the initiation of EC use during pregnancy does not typically lead to CC abstinence, an important finding that differs from intervention studies.^9^

Our finding that EC initiators, relative to quitters, were more likely to have demographic risk factors and less likely to have the benefit of protective health care and behaviors is broadly consistent with prior surveys.^22^ Novel findings include the fact that EC initiators were more likely than both CC-exclusive users and quitters to be heavy smokers and have depression, but were less likely to be obese. Understanding who is most likely to initiate EC use could inform targeted interventions aimed at helping smokers use the safest and most empirically supported interventions.

Our findings reinforce the well-established evidence that the offspring of pregnant individuals who smoke CCs in pregnancy are at elevated risk for neonatal health complications. Our results are less certain when comparing EC initiators to quitters and CC-exclusive users. Consistent with prior literature, point estimates for the risk among EC initiators tended to fall between those of quitters and CC-exclusive users.^11,14^ However, our analyses did not provide sufficient evidence to conclude that EC use and associated CC reduction were associated with lower risk of adverse perinatal outcomes. Owing to small sample sizes, we had limited ability to evaluate differential outcomes of dual users and EC replacers. However, dual users, who account for 70% of EC initiators, appear to be at elevated risk for SGA, with a prevalence similar to CC-exclusive users, whereas EC replacers had notably lower risk that was more consistent with quitters. This is consistent with prior PRAMS scholarship and may reflect a CC-specific link to SGA.^11,14,23,24^

A unique contribution is the finding that EC initiators generally and dual users specifically reduced CC use during and after pregnancy relative to prepregnancy levels more than CC-exclusive users. It remains plausible, therefore, that EC initiation aids in CC use reduction even if most EC initiators do not quit CCs. However, our study was not designed to estimate the causal effect of EC initiation on health behaviors and outcomes. Differences in the number of CCs smoked could be attributable to confounding influences (e.g., preexisting differences in motivation to quit smoking) which were not measured in PRAMS.

The U.S. CDC advises that EC initiation in the general adult nonpregnant population should be considered a viable cessation strategy only if it results in CC abstinence.^1^ Clinicians should caution patients that EC initiators appear more likely to become dual users than EC-exclusive users. Furthermore, ECs, particularly nicotine-containing ECs, may be harmful during pregnancy, and there is currently no compelling evidence of a causal effect of EC initiation on levels of CC use in pregnancy.^11^ Our findings, in combination with prior scholarship, reinforce current public health guidelines advising against the use of ECs as a prenatal smoking cessation tool.^1,5^

### Strengths and Limitations

This study had several limitations. First, it relied on self-reported cigarette use in the 3 months prior to pregnancy, the last 3 months of pregnancy, and 2–6 months post-delivery. Data on women’s cigarette use early in pregnancy are not available. Misclassification of women’s pattern of cigarette use is thus likely. Furthermore, recall bias, particularly for prepregnancy cigarette use is likely given the survey is completed after delivery. However, past studies suggest self-reported smoking is fairly accurate and well represents perinatal smoking patterns.^25^ Underreporting of tobacco use on self-report measures varies across populations (eg, racial groups), which could lead to underestimates of associations between smoking exposure and health outcomes and either under or overestimates of associations with participant characteristics, depending on the nature of the biased reporting.^26^ Second, we assumed that EC initiators started using ECs to aid in CC smoking cessation. While we cannot confirm this, it seems unlikely that CC smokers would choose pregnancy as the time to begin recreational EC use. PRAMS does not systematically capture data regarding the use of other smoking cessation strategies or intention/willingness to quit smoking. Consequently, we could not distinguish between pregnant smokers initiating EC use as a cessation strategy versus those who intend to substitute ECs for CCs. Third, as PRAMS did not capture the amount of EC use (only frequency), we could not evaluate total CC/EC tobacco consumption. Fourth, PRAMS did not assess for use of nicotine-containing products other than CCs and ECs that potentially contribute to adverse pregnancy outcomes, though these products are uncommonly used in pregnancy.^27^ Fifth, given the design of the survey, we did not attempt to draw causal conclusions. Sixth, there were relatively few participants reporting exclusive EC use in late pregnancy, limiting our ability to make statistical comparisons with this group. Finally, we adjusted for race/ethnicity as a marker of influential social processes (eg, broad cultural differences and differential exposure to discrimination) that were not measured directly. We do not, however, view race/ethnicity as a construct that inherently modulates risk.^28^ Ideally, future research would explicitly account for differences in social experiences across race and ethnic groups rather than relying on race/ethnicity as an imprecise proxy. Additional limitations include our restriction to women with live singleton births although we do not have reason to believe that the pattern of EC initiation among smokers varies by plurality and pregnancy outcomes. Overall, these limitations were outweighed by important strengths. Chief among those was having a large dataset from a national surveillance study and the use of analysis weights intended to improve the sample’s ability to represent the broader U.S. population.

## Conclusions

Only a small proportion of U.S. pregnant smokers report smoking ECs during the last 3 months of pregnancy, and most who do so also smoke CCs. Healthcare workers should be cognizant of the accumulating real-world evidence that CC cessation does not appear to be the typical outcome of prenatal EC initiation. The fact that > 40% of pregnant smokers in this national study continued smoking CCs underscores the scope of this public health threat. Improving the delivery of established evidence-based interventions to promote tobacco abstinence in pregnancy must be a national priority.

## Supplementary material

Supplementary material is available at *Nicotine and Tobacco Research* online.

ntae119_suppl_Supplementary_Figures

ntae119_suppl_Supplementary_Tables

## Data Availability

The data underlying the results presented in the study are available from the Centers for Disease Control and Prevention Pregnancy Risk Assessment Monitory System.
