# Supplementary material for: Demographic Characteristics, Perinatal Smoking Patterns, and Risk for Neonatal Health Complications Among Pregnant Smokers in the United States Who Begin Using Electronic Cigarettes During Pregnancy: A Descriptive Study Using Population-Based Surveillance Data
Source: Nicotine Tob Res. 2024 May 23;26(11):1455–62. doi: 10.1093/ntr/ntae119 (PMC11494498; doi:10.1093/ntr/ntae119)
Supplement: ntae119_suppl_Supplementary_Figures [file ntae119_suppl_supplementary_figures.pdf]

Figure S1.

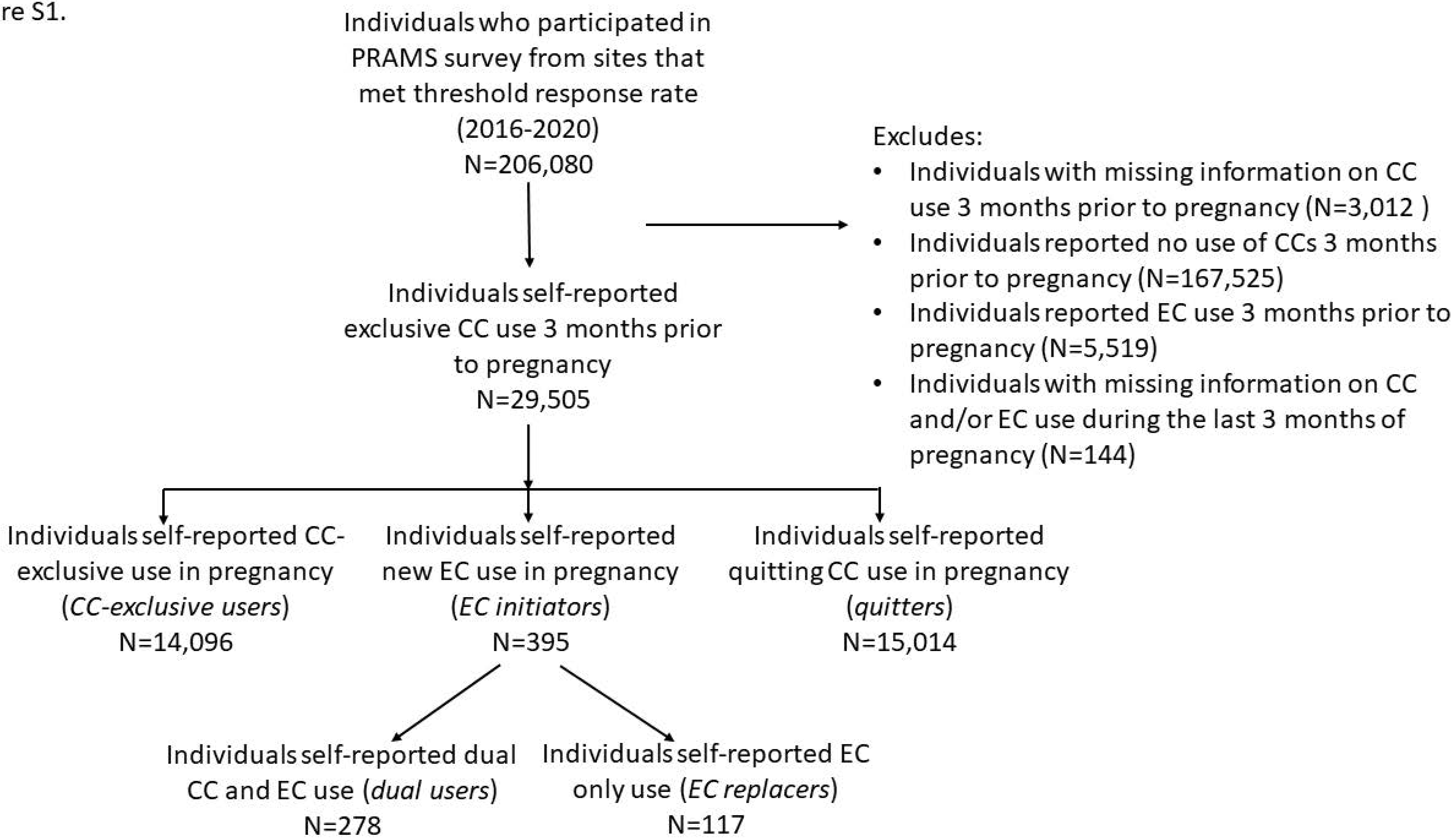

Individuals who participated in PRAMS survey from sites that met threshold response rate (2016-2020)
N=206,080
Excludes:
• Individuals with missing information on CC use 3 months prior to pregnancy (N=3,012)
• Individuals reported no use of CCs 3 months prior to pregnancy (N=167,525)
• Individuals reported EC use 3 months prior to pregnancy (N=5,519)
• Individuals with missing information on CC and/or EC use during the last 3 months of pregnancy (N=144)
Individuals self-reported exclusive CC use 3 months prior to pregnancy
N=29,505
Individuals self-reported CC-exclusive use in pregnancy (*CC-exclusive users*)
N=14,096
Individuals self-reported new EC use in pregnancy (*EC initiators*)
N=395
Individuals self-reported quitting CC use in pregnancy (*quitters*)
N=15,014
Individuals self-reported dual CC and EC use (*dual users*)
N=278
Individuals self-reported EC only use (*EC replacers*)
N=117

Figure S2.

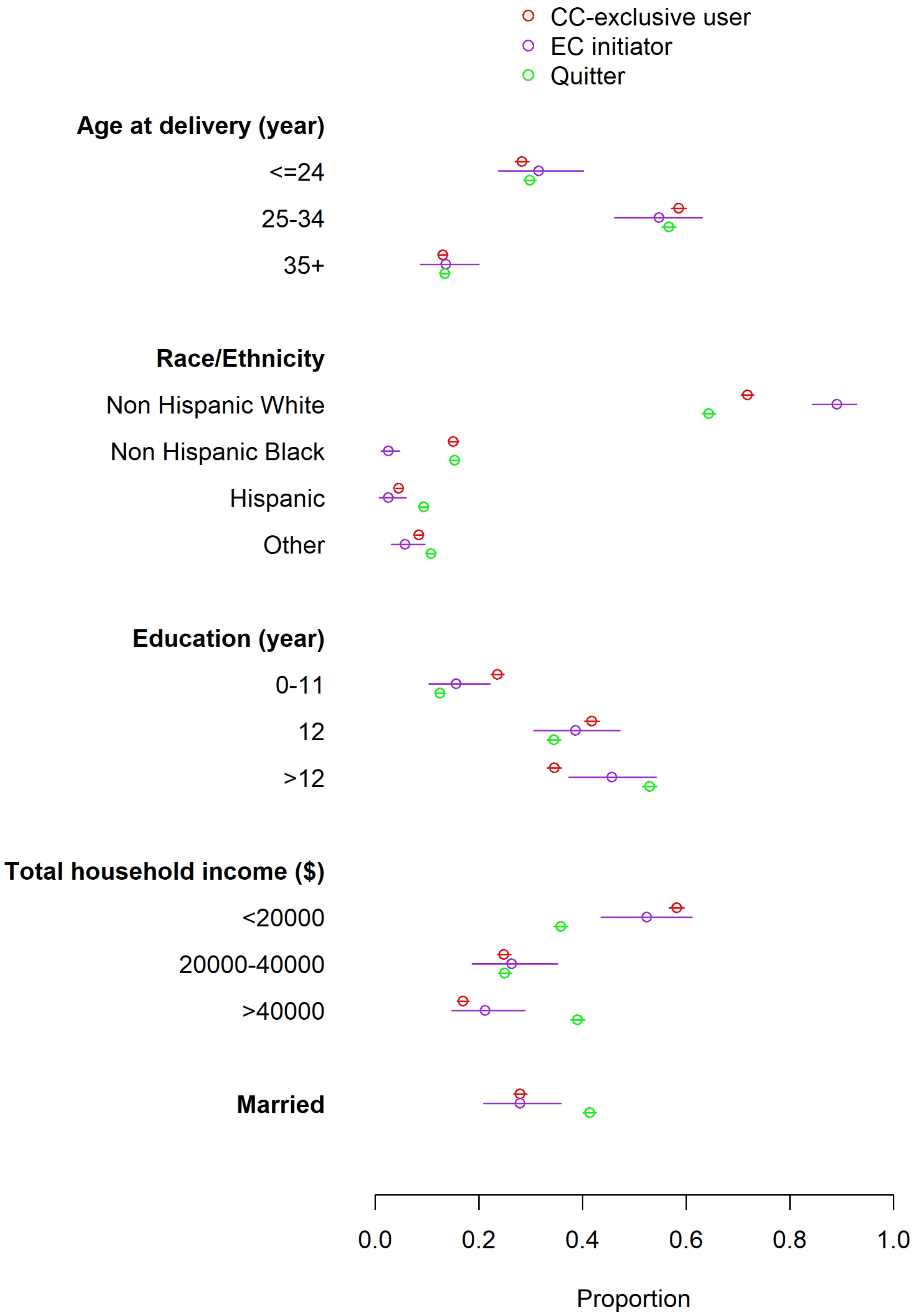
CC-exclusive user
EC initiator
Quitter
Age at delivery (year)
<=24
25-34
35+
Race/Ethnicity
Non Hispanic White
Non Hispanic Black
Hispanic
Other
Education (year)
0-11
12
>12
Total household income ($)
<20000
20000-40000
>40000
Married
0.0
0.2
0.4
0.6
0.8
1.0
Proportion

Figure S3

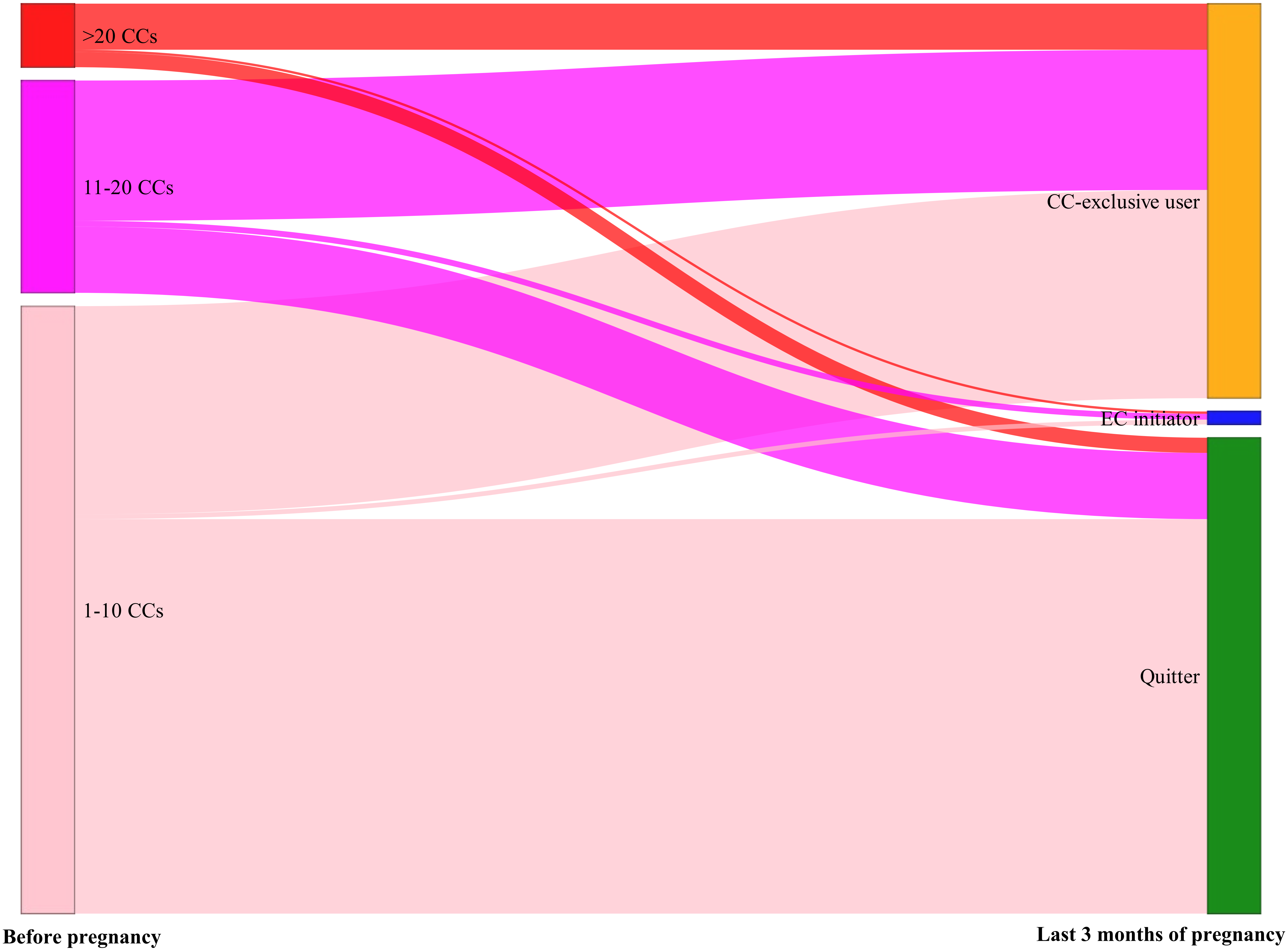
>20 CCs
11-20 CCs
1-10 CCs
CC-exclusive user
EC initiator
Quitter
Before pregnancy
Last 3 months of pregnancy

Figure S4.

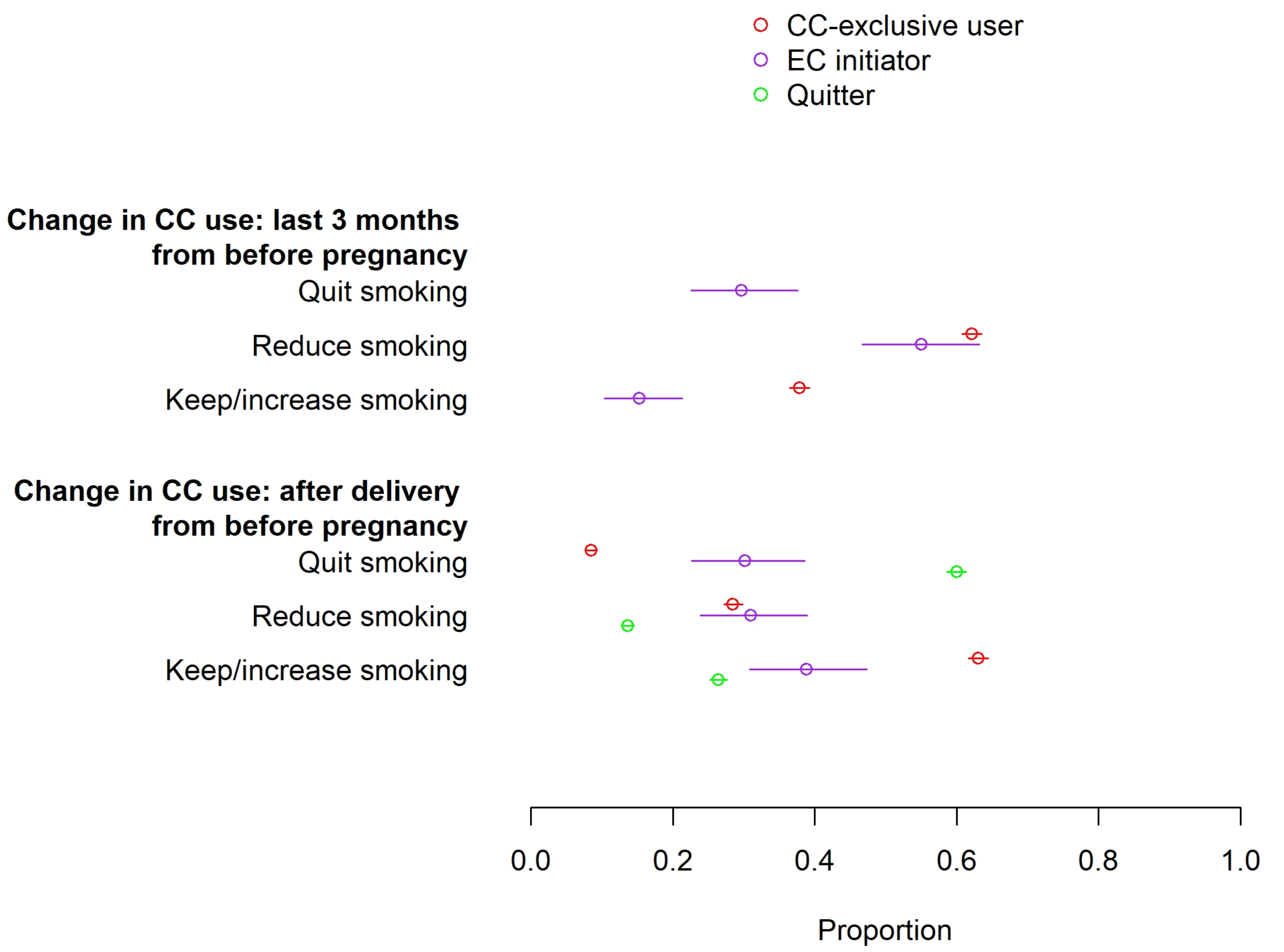
CC-exclusive user
EC initiator
Quitter
Change in CC use: last 3 months
from before pregnancy
Quit smoking
Reduce smoking
Keep/increase smoking
Change in CC use: after delivery
from before pregnancy
Quit smoking
Reduce smoking
Keep/increase smoking
0.0
0.2
0.4
0.6
0.8
1.0
Proportion

Figure S5.

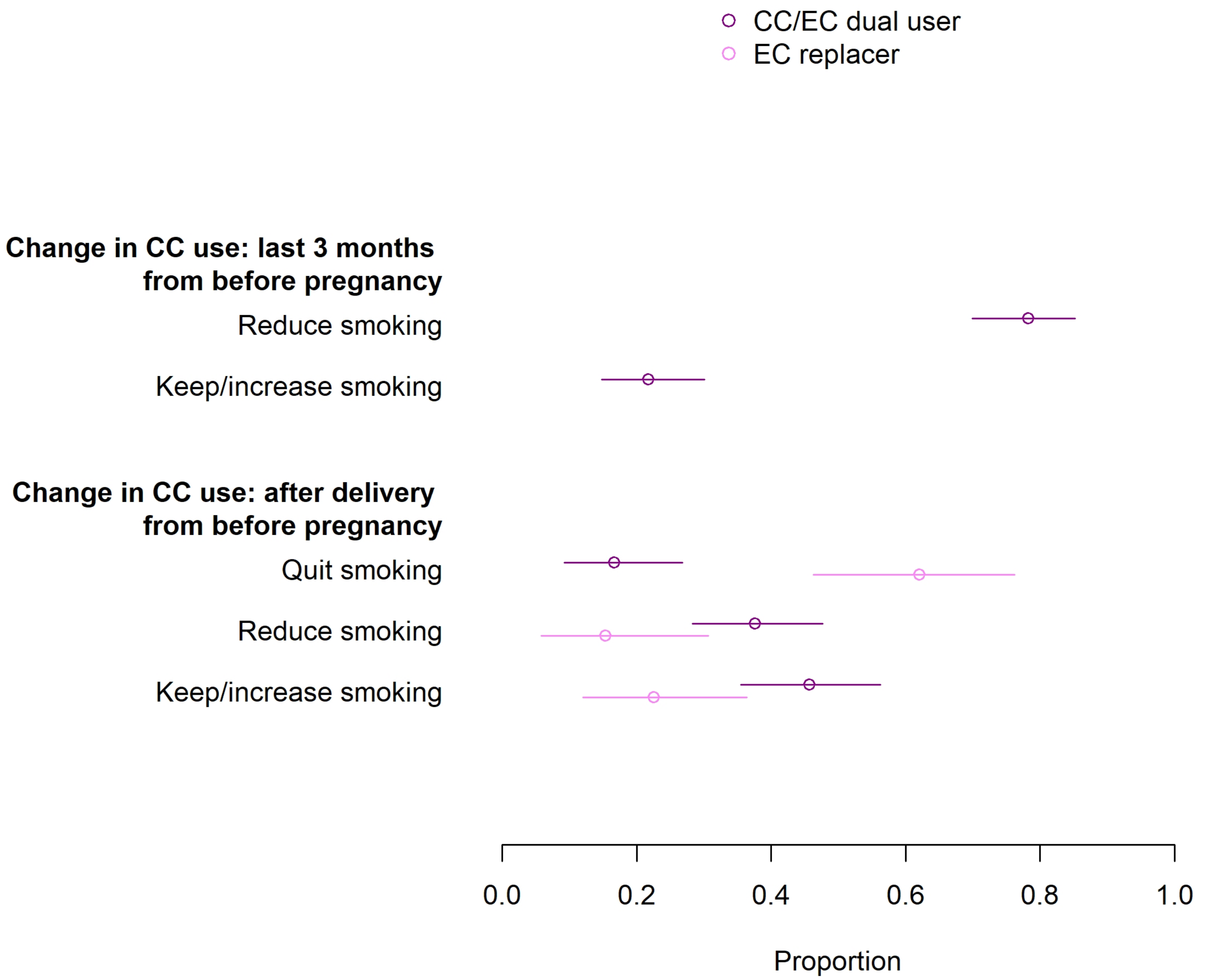
CC/EC dual user
EC replacer
Change in CC use: last 3 months from before pregnancy
Reduce smoking
Keep/increase smoking
Change in CC use: after delivery from before pregnancy
Quit smoking
Reduce smoking
Keep/increase smoking
0.0
0.2
0.4
0.6
0.8
1.0
Proportion

Figure S6.

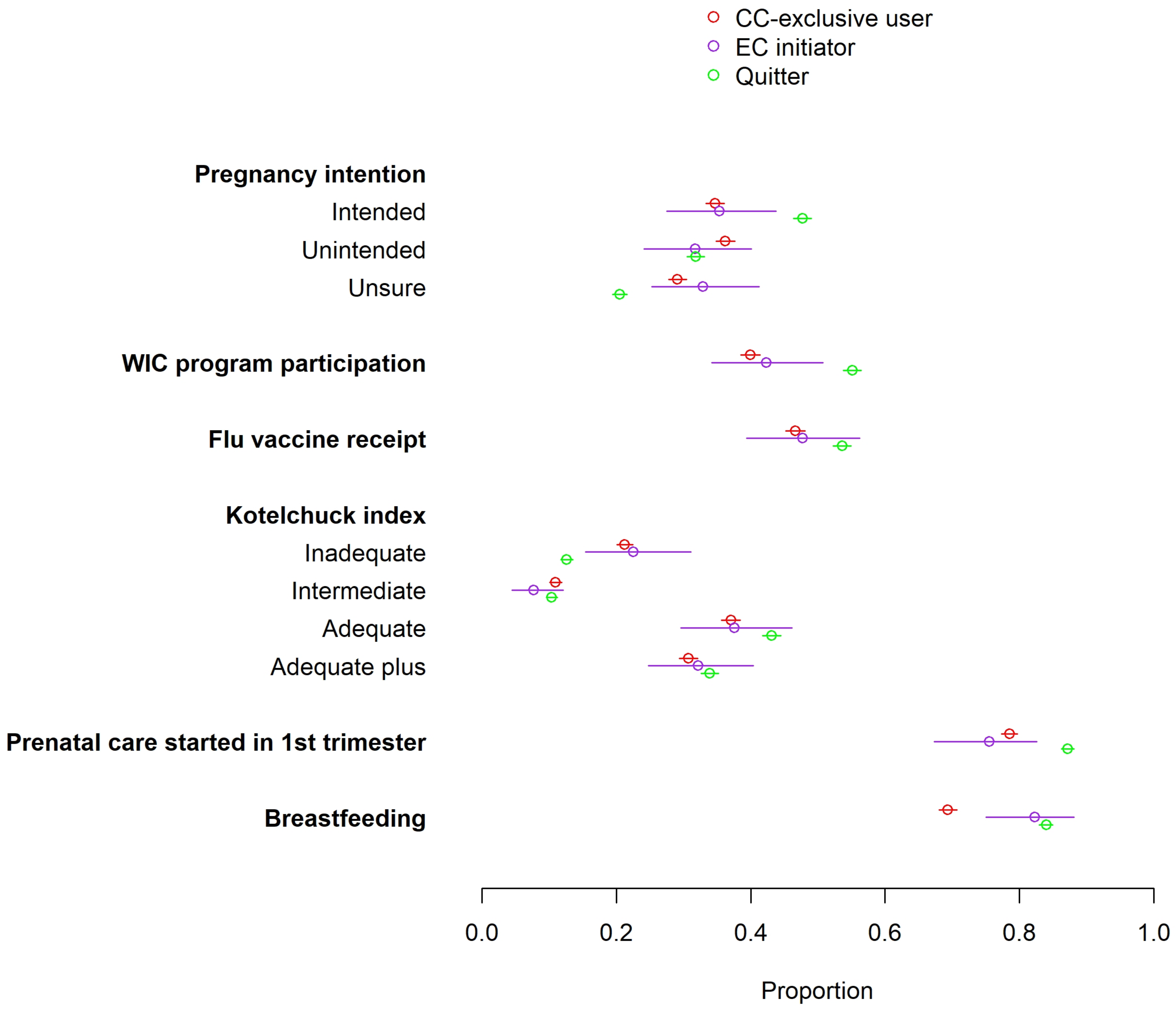

CC-exclusive user
EC initiator
Quitter
Pregnancy intention
Intended
Unintended
Unsure
WIC program participation
Flu vaccine receipt
Kotelchuck index
Inadequate
Intermediate
Adequate
Adequate plus
Prenatal care started in 1st trimester
Breastfeeding
0.0
0.2
0.4
0.6
0.8
1.0
Proportion

Figure S7.

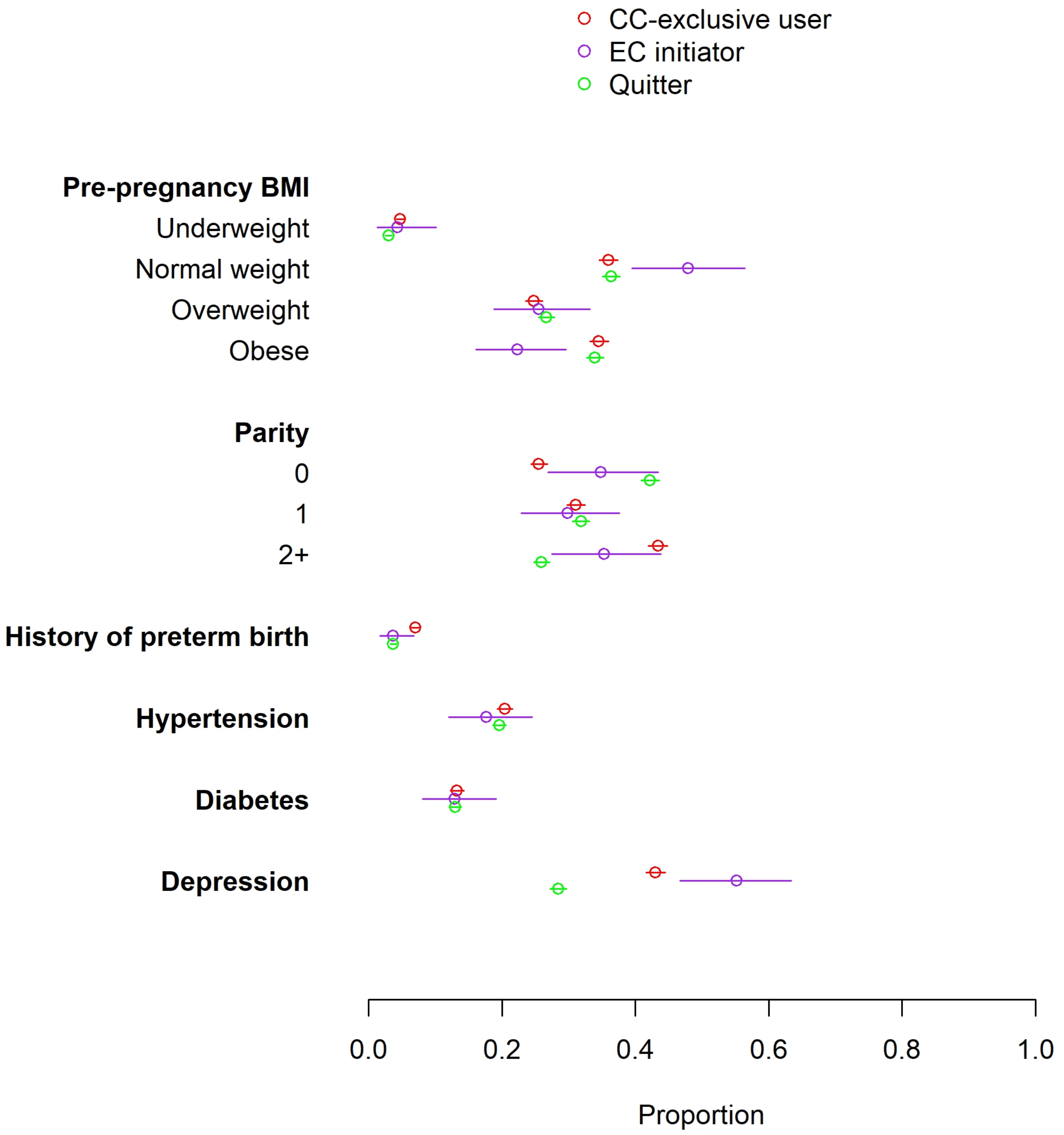
CC-exclusive user
EC initiator
Quitter
Pre-pregnancy BMI
Underweight
Normal weight
Overweight
Obese
Parity
0
1
2+
History of preterm birth
Hypertension
Diabetes
Depression
0.0
0.2
0.4
0.6
0.8
1.0
Proportion

Figure S8.

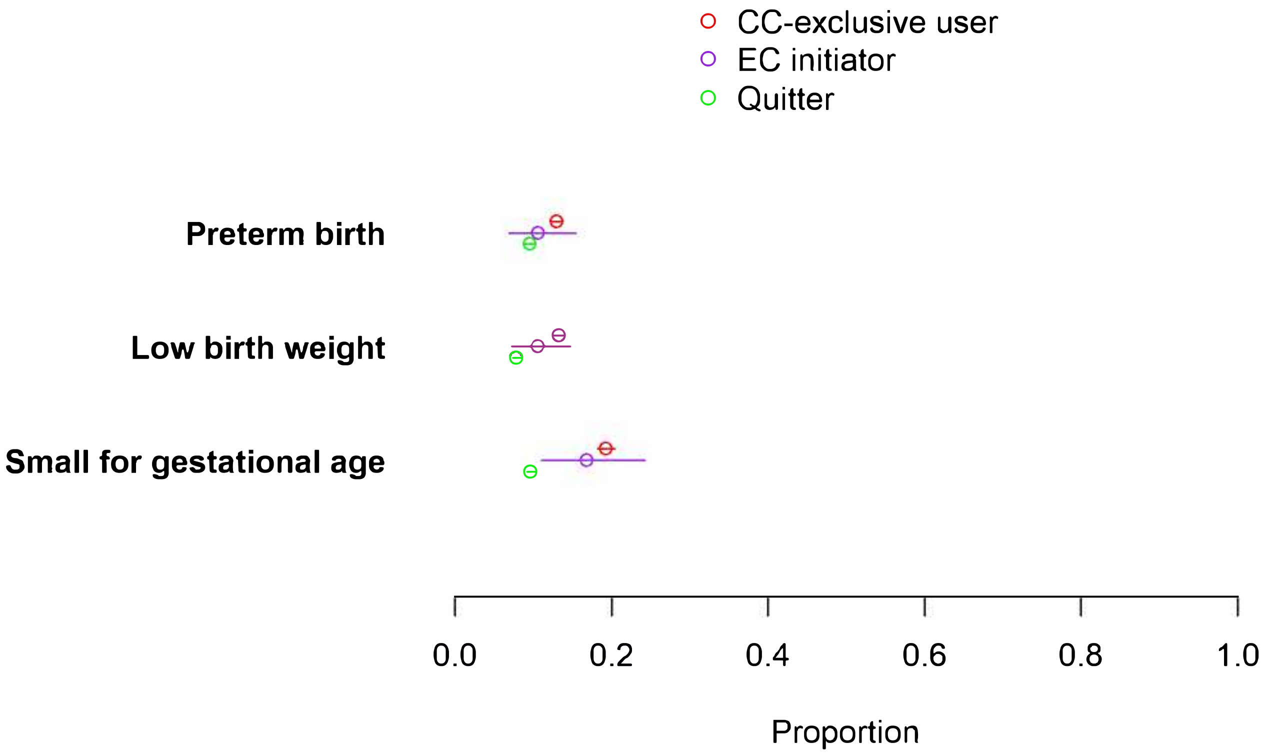

CC-exclusive user
EC initiator
Quitter
Preterm birth
Low birth weight
Small for gestational age
0.0
0.2
0.4
0.6
0.8
1.0
Proportion

Figure S9.

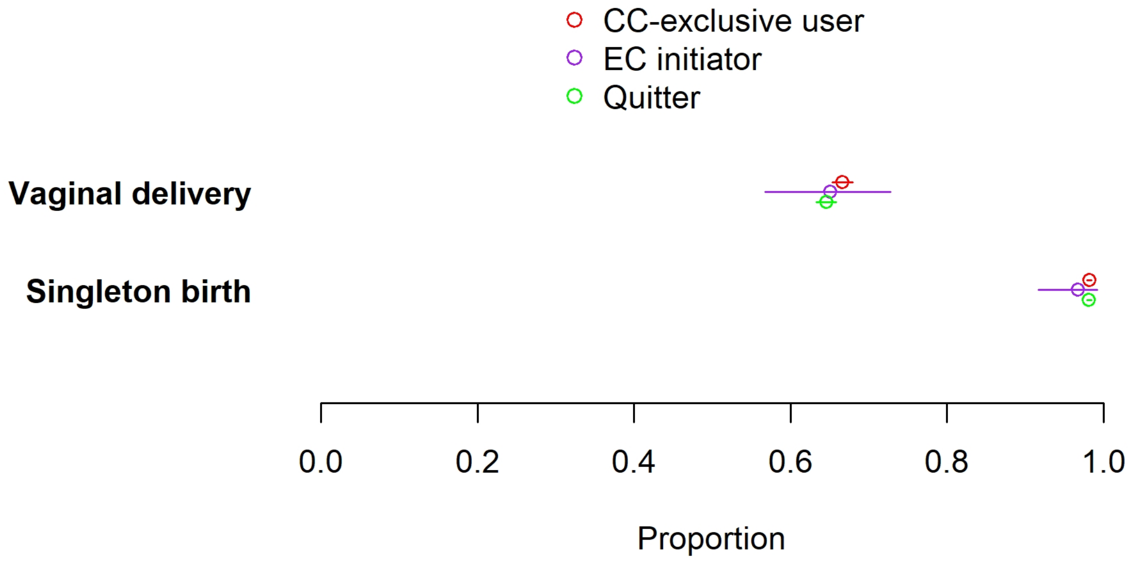

CC-exclusive user
EC initiator
Quitter
Vaginal delivery
Singleton birth
0.0
0.2
0.4
0.6
0.8
1.0
Proportion

Figure S10.

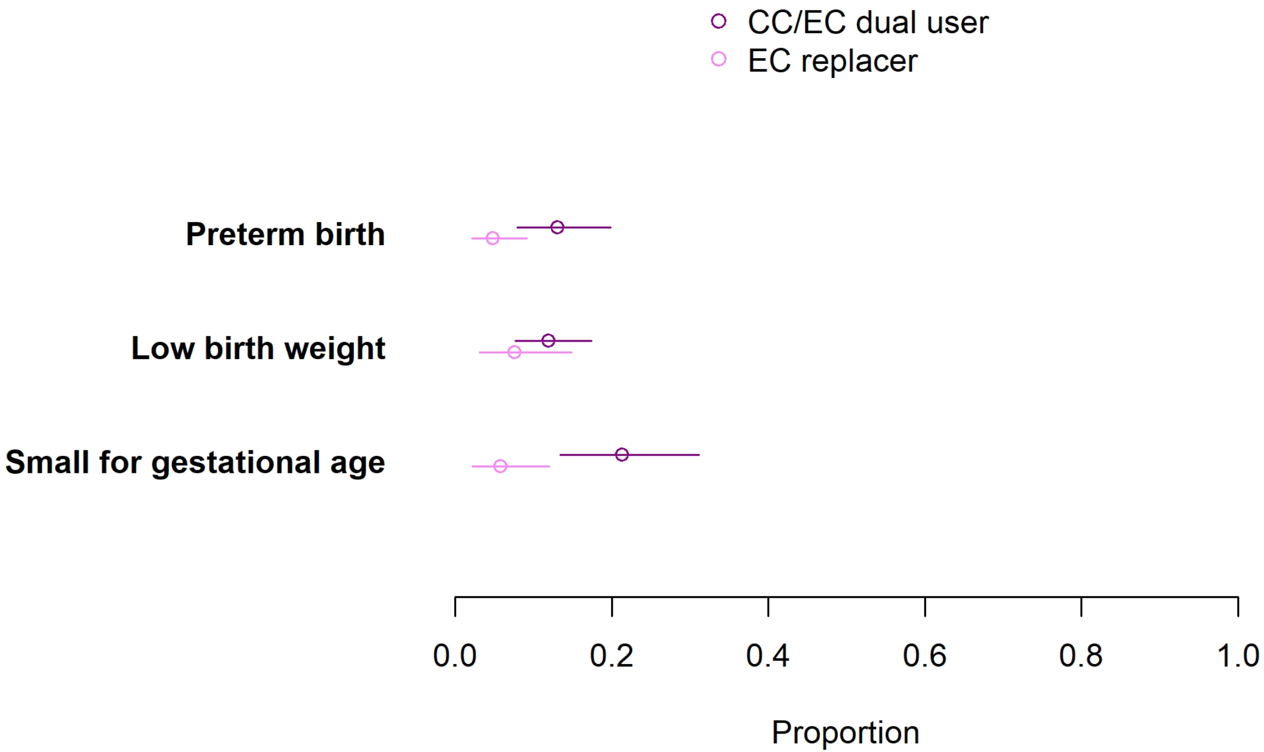

CC/EC dual user
EC replacer
Preterm birth
Low birth weight
Small for gestational age
0.0
0.2
0.4
0.6
0.8
1.0
Proportion
