# Supplementary material for: Demographic Characteristics, Perinatal Smoking Patterns, and Risk for Neonatal Health Complications Among Pregnant Smokers in the United States Who Begin Using Electronic Cigarettes During Pregnancy: A Descriptive Study Using Population-Based Surveillance Data
Source: Nicotine Tob Res. 2024 May 23;26(11):1455–62. doi: 10.1093/ntr/ntae119 (PMC11494498; doi:10.1093/ntr/ntae119)
Supplement: ntae119_suppl_Supplementary_Tables [file ntae119_suppl_supplementary_tables.docx]

Demographic characteristics, perinatal smoking patterns, and risk for neonatal health complications among pregnant smokers in the United States who begin using electronic cigarettes during pregnancy: A descriptive study using population-based surveillance data

**Online supplement**

**Table of Contents**

[Table S1 2](#_Toc160784501)

[Table S2 3](#_Toc160784502)

[Table S3 4](#_Toc160784503)

[Table S4 5](#_Toc160784504)

[Table S5 6](#_Toc160784505)

[Table S6 7](#_Toc160784506)

[Table S7 8](#_Toc160784507)

[**Supplemental Figure Legends** 9](#_Toc160784508)

Table S1. Measurement of cigarette use in the PRAMS Survey, 2016-2020.

| **Combustible Cigarette Use** | |
| --- | --- |
| Have you smoked any cigarettes in the past 2 years? | No (skip follow-up questions)  ***Yes*** (continue to follow-up questions) |
| In the 3 months before you got pregnant, how many cigarettes did you smoke on an average day? | - I didn’t smoke then. - Less than 1 cigarette - 1 to 5 cigarettes - 6 to 10 cigarettes - 11 to 20 cigarettes - 21 to 40 cigarettes   41 cigarettes of more |
| In the last 3 months of your pregnancy, how many cigarettes did you smoke on an average day? |  |
| How many cigarettes do you smoke on an average day now? |  |
| **Electronic Cigarette & Nicotine Products Use** | |
| Have you used e-cigarettes or other electronic nicotine products in the past 2 years? | No (skip follow-up questions)  ***Yes*** (continue to follow-up questions) |
| During the 3 months before you got pregnant, on average, how often did you use e-cigarettes or other electronic nicotine products? | - I did not use e-cigarettes or other electronic nicotine products then - 1 day a week or less - 2-6 days a week - Once a day   More than once a day |
| During the last 3 months of your pregnancy, on average, how often did you use e-cigarettes or other electronic nicotine products? |  |

PRAMS, Pregnancy Risk Assessment Monitoring System

Table S2. Daily average amount of combustible cigarette usage before, during, and after pregnancy of smokers who initiated e-cigarettes during pregnancy, PRAMS, 2016-2020

| Average daily use | CC/EC dual user | | | EC replacer | | |
| --- | --- | --- | --- | --- | --- | --- |
|  | N^a^ | % (95% CI)^b^ | RSE (%)^c^ | N | % (95% CI) | RSE (%) |
| Before pregnancy | 278 |  |  | 117 |  |  |
| 0 cigarette | - | - | - | - | - | - |
| 1-<10 cigarettes | 98 | 32.9 (23.4, 43.5) | 14.8 | 60 | 45.5 (30.5, 61.1) | 15.6 |
| 10-20 cigarettes | 126 | 48.6 (38.3, 59.1) | 10.4 | 44 | 37.8 (23.4, 54.0) | 18.7 |
| >20 cigarettes | 54 | 18.5 (11.7, 27.0) | 19.9 | 13 | 16.7 (6.2, 33.3) | 36.1 |
| During pregnancy | 278 |  |  | 117 |  |  |
| 0 cigarette | 0 | 0 | - | 117 | 100 | - |
| 1-<10 cigarettes | 225 | 81.4 (73.0, 88.0) | 4.4 | 0 | 0 | - |
| 10-20 cigarettes | 40 | 14.2 (8.4, 21.9) | 22.6 | 0 | 0 | - |
| >20 cigarettes | 13 | 4.4 (1.6, 9.6) | 41.0 | 0 | 0 | - |
| After pregnancy | 277 |  |  | 117 |  |  |
| 0 cigarette | 35 | 16.7 (9.2, 26.8) | 25.1 | 73 | 62.1 (46.3, 76.2) | 11.1 |
| 1-<10 cigarettes | 141 | 47.4 (37.1, 57.8) | 10.6 | 32 | 29.8 (16.9, 45.6) | 22.1 |
| 10-20 cigarettes | 76 | 27.5 (19.0, 37.4) | 16.1 | 11 | 7.7 (2.3, 18.0) | 43.8 |
| >20 cigarettes | 25 | 8.5 (4.0, 15.4) | 31.1 | 1 | 0.4 (0.0, 2.4) | 98.7 |

^a^Unweighted sample size; ^b^Weighted prevalence and 95% confidence interval. ^c^Relative standard error of weighted prevalence with higher value indicates statistical unreliable estimation. Statistical analysis by a Rao-Scott Chi-square test.

Table S3. Multivariable logistic regression analyses* of having preterm birth, low birth weight, and SGA comparing EC initiators to CC exclusive users and quitters, PRAMS, 2016-2020

| Outcome | EC initiator vs. CC exclusive user | | EC initiator vs. quittor | |
| --- | --- | --- | --- | --- |
|  | aOR (95%CI) | P value | aOR (95%CI) | P value |
| Preterm birth | 0.97 (0.59, 1.61) | 0.91 | 1.18 (0.71, 1.96) | 0.53 |
| Low birth weight | 0.70 (0.41, 1.21) | 0.20 | 1.52 (0.88, 2.61) | 0.14 |
| SGA | 0.82 (0.52, 1.29) | 0.39 | 1.42 (0.90,2.25) | 0.13 |

*Models were adjusted for maternal age, race/ethnicity, education level, household income, marital status, prenatal participation in the WIC program, pregnancy intention, flu vaccine receipt, the Kotelchuck index, initiation of prenatal care in the first trimester, parity, history of preterm birth, maternal pre-pregnancy BMI, pre-existing and/or gestational hypertension, pre-existing and/or gestational diabetes, self-reported diagnosis of depression before and/or during pregnancy, and year of delivery.

Table S4. Perinatal and neonatal health outcomes of EC initiators who became dual users or EC replacers, PRAMS, 2016-2020

| Average daily use | CC/EC dual user | | | EC replacer | | |
| --- | --- | --- | --- | --- | --- | --- |
|  | N^a^ | % (95% CI)^b^ | RSE (%)^c^ | N | % (95% CI) | RSE (%) |
| Delivery method | 277 |  |  | 117 |  |  |
| Vaginal | 175 | 65.5 (55.2, 74.7) | 7.3 | 76 | 64.2 (47.8, 78.5) | 11.0 |
| C-section | 102 | 34.5 (25.3, 44.8) | 13.7 | 41 | 35.8 (21.5, 52.2) | 19.8 |
| Plurality | 257 |  |  | 115 |  |  |
| Singleton | 248 | 98.5 (95.2, 99.7) | 1.0 | 110 | 92.8 (75.2, 99.2) | 5.3 |
| More than one | 9 | 1.5 (0.3, 4.8) | 61.8 | 5 | 7.2 (0.8, 24.8) | 67.9 |
| Preterm birth | 277 |  |  | 117 |  |  |
| No | 204 | 86.9 (80.2, 92.1) | 3.3 | 97 | 95.2 (90.8, 97.9) | 1.6 |
| Yes | 73 | 13.1 (7.9, 19.8) | 21.8 | 20 | 4.8 (2.1, 9.2) | 32.5 |
| Low birth weight | 278 |  |  | 117 |  |  |
| No | 178 | 88.1 (82.6, 92.4) | 2.7 | 92 | 92.5 (85.1, 96.9) | 2.8 |
| Yes | 100 | 11.9 (7.6, 17.4) | 19.7 | 25 | 7.5 (3.1, 14.9) | 34.3 |
| Small-for-gestational-age (SGA) | 267 |  |  | 112 |  |  |
| No | 197 | 78.7 (68.8, 86.6) | 5.4 | 96 | 94.3 (88.0, 97.8) | 2.3 |
| Yes | 70 | 21.3 (13.4, 31.2) | 20.1 | 16 | 5.7 (2.2, 12.0) | 37.1 |

^a^Unweighted sample size; ^b^Weighted prevalence and 95% confidence interval. ^c^Relative standard error of weighted prevalence with higher value indicates statistical unreliable estimation. Statistical analysis by a Rao-Scott Chi-square test.

Table S5. Participant demographic characteristics of EC initiators who became dual users or EC replacers, PRAMS, 2016-2020

|  | CC/EC dual user | | | EC replacer | | |
| --- | --- | --- | --- | --- | --- | --- |
|  | N^a^ | % (95% CI)^b^ | RSE (%)^c^ | N | % (95% CI) | RSE (%) |
| Maternal age | 278 |  |  | 117 |  |  |
| <=24 | 76 | 28.7 (19.8, 38.9) | 16.1 | 37 | 38.4 (23.2, 55.5) | 19.4 |
| 25-34 | 164 | 57.6 (47.2, 67.6) | 8.6 | 65 | 48.0 (32.8, 63.4) | 14.8 |
| 35+ | 38 | 13.7 (8.0, 21.4) | 23.4 | 15 | 13.6 (5.0, 27.9) | 37.1 |
| Maternal race/ethnicity | 276 |  |  | 116 |  |  |
| Non-Hispanic White | 218 | 91.8 (86.8, 95.3) | 2.2 | 83 | 82.8 (70.0, 91.7) | 5.9 |
| Non-Hispanic Black | 14 | 2.3 (0.8, 5.4) | 43.9 | 8 | 3.0 (0.9, 7.2) | 44.0 |
| Hispanic | 8 | 1.9 (0.3, 6.0) | 62.9 | 6 | 4.2 (0.6, 13.9) | 63.8 |
| Other | 36 | 4.0 (1.9, 7.4) | 31.7 | 19 | 10.0 (3.3, 21.9) | 40.5 |
| Maternal education | 277 |  |  | 117 |  |  |
| Some high school or less | 47 | 17.1 (10.5, 25.6) | 21.1 | 16 | 12.1 ( 4.6, 24.4) | 36.1 |
| High school graduate | 116 | 39.7 (29.9, 50.2) | 12.4 | 35 | 36.1 (21.5, 52.9) | 20.1 |
| Some college or more | 114 | 43.2 (33.0, 53.8) | 11.7 | 66 | 51.8 (36.0, 67.2) | 14.0 |
| Household income | 261 |  |  | 111 |  |  |
| < $20,000 | 165 | 60.9 (49.9, 71.2) | 8.5 | 49 | 32.5 (19.9, 47.3) | 19.3 |
| $20,000 - $40,000 | 57 | 22.5 (14.2, 32.8) | 19.9 | 30 | 35.3 (19.7, 53.6) | 22.1 |
| >40,000 | 39 | 16.5 ( 9.3, 26.2) | 24.3 | 32 | 32.2 (19.2, 47.6) | 20.3 |
| Marital status | 278 |  |  | 117 |  |  |
| Married | 68 | 24.2 (16.2, 33.8) | 17.6 | 40 | 37.0 (23.1, 52.7) | 18.5 |
| Other | 210 | 75.8 (66.2, 83.8) | 5.6 | 77 | 63.0 (47.3, 76.9) | 10.9 |
| Year of delivery | 278 |  |  | 117 |  |  |
| 2016 | 54 | 19.8 (12.4, 29.1) | 20.3 | 22 | 13.4 ( 5.4, 26.1) | 34.5 |
| 2017 | 51 | 13.1 ( 8.1, 19.7) | 21.2 | 21 | 20.6 (10.3, 34.8) | 27.0 |
| 2018 | 58 | 24.7 (16.2, 35.0) | 18.4 | 25 | 25.0 (13.5, 39.8) | 24.0 |
| 2019 | 57 | 21.7 (13.7, 31.5) | 19.7 | 27 | 21.8 (10.7, 36.8) | 27.2 |
| 2020 | 58 | 20.7 (13.1, 30.3) | 20.0 | 22 | 19.2 ( 7.7, 36.4) | 33.5 |

^a^Unweighted sample size; ^b^Weighted prevalence and 95% confidence interval. ^c^Relative standard error of weighted prevalence with higher value indicates statistical unreliable estimation. Statistical analysis by a Rao-Scott Chi-square test.

Table S6. Participant health behaviors of EC initiators who became dual users and EC replacers, PRAMS, 2016-2020

|  | CC/EC dual user | | | EC replacer | | |
| --- | --- | --- | --- | --- | --- | --- |
|  | N^a^ | % (95% CI)^b^ | RSE (%)^c^ | N | % (95% CI) | RSE (%) |
| Pregnancy intention | 271 |  |  | 115 |  |  |
| Intended | 89 | 34.0 (24.4, 44.6) | 14.5 | 50 | 38.7 (24.7, 54.1) | 17.6 |
| Unintended | 95 | 28.9 (20.2, 38.8) | 15.6 | 41 | 38.7 (23.5, 55.7) | 19.2 |
| Not sure | 87 | 37.1 (27.5, 47.5) | 13.1 | 24 | 22.6 (11.2, 38.1) | 27.0 |
| WIC program participation | 274 |  |  | 116 |  |  |
| No | 165 | 61.0 (50.6, 70.7) | 8.0 | 56 | 49.8 (34.2, 65.5) | 14.5 |
| Yes | 109 | 39.0 (29.3, 49.4) | 12.6 | 60 | 50.2 (34.5, 65.8) | 14.4 |
| Flu vaccine receipt | 274 |  |  | 117 |  |  |
| No | 149 | 56.6 (46.0, 66.8) | 9.0 | 47 | 42.0 (26.9, 58.4) | 17.3 |
| Yes | 125 | 43.4 (33.2, 54.0) | 11.7 | 70 | 58.0 (41.6, 73.1) | 12.6 |
| Kotelchuck index | 274 |  |  | 114 |  |  |
| Inadequate | 59 | 26.6 (17.4, 37.6) | 18.3 | 17 | 12.9 ( 4.3, 27.9) | 40.2 |
| Intermediate | 26 | 8.7 (4.6, 14.7) | 27.1 | 8 | 5.2 (1.3, 13.3) | 48.3 |
| Adequate | 96 | 37.2 (27.4, 48.0) | 13.5 | 44 | 38.4 (24.3, 54.1) | 17.9 |
| Adequate plus | 93 | 27.4 (19.3, 36.9) | 15.6 | 45 | 43.5 (28.1, 59.9) | 17.0 |
| Prenatal care started in the first trimester of pregnancy | 272 |  |  | 115 |  |  |
| No/no prenatal care | 66 | 27.3 (18.5, 37.7) | 17.0 | 24 | 17.9 (7.4, 33.7) | 32.9 |
| Yes | 206 | 72.7 (62.3, 81.5) | 6.4 | 91 | 82.1 (66.3, 92.6) | 7.2 |
| Breastfeeding | 263 |  |  | 112 |  |  |
| Never | 54 | 19.2 (11.9, 28.5) | 20.7 | 17 | 14.2 (5.5, 28.0) | 35.2 |
| Ever | 209 | 80.8 (71.5, 88.1) | 4.9 | 95 | 85.8 (72.0, 94.5) | 5.9 |

^a^Unweighted sample size; ^b^Weighted prevalence and 95% confidence interval. ^c^Relative standard error of weighted prevalence with higher value indicates statistical unreliable estimation. Statistical analysis by a Rao-Scott Chi-square test.

Table S7. Participant health conditions of EC initiators who became dual users and EC replacers, PRAMS, 2016-2020

|  | CC/EC dual user | | | EC replacer | | |
| --- | --- | --- | --- | --- | --- | --- |
|  | N^a^ | % (95% CI)^b^ | RSE (%)^c^ | N^a^ | % (95% CI)^b^ | RSE (%) |
| Pre-pregnancy BMI | 274 |  |  | 113 |  |  |
| Underweight (<18.5kg/m^2)^ | 15 | 4.7 (0.9, 13.4) | 57.2 | 6 | 3.4 (0.5, 10.7) | 61.0 |
| Normal (18.5-24.9kg/m^2^) | 126 | 46.3 (36.2, 56.7) | 10.8 | 48 | 51.6 (35.8, 67.1) | 14.1 |
| Overweight (25.0-29.9kg/m^2^) | 67 | 27.3 (18.8, 37.1) | 16.3 | 31 | 21.5 (11.5, 34.6) | 24.5 |
| Obese (>=30.0kg/m^2^) | 66 | 21.7 (14.3, 30.7) | 18.2 | 28 | 23.6 (12.5, 38.3) | 24.8 |
| Parity | 277 |  |  | 117 |  |  |
| Primiparous | 90 | 32.8 (23.2, 43.6) | 15.0 | 42 | 39.5 (24.8, 55.7) | 18.1 |
| 2 | 82 | 26.2 (18.5, 35.2) | 15.5 | 38 | 38.5 (24.0, 54.6) | 18.4 |
| 3+ | 105 | 41.0 (30.9, 51.7) | 12.3 | 37 | 22.0 (11.6, 35.9) | 25.1 |
| History of preterm birth | 275 |  |  | 117 |  |  |
| No | 255 | 95.7 (91.5, 98.2) | 1.6 | 114 | 97.8 (92.0, 99.8) | 1.6 |
| Yes | 20 | 4.3 (1.8, 8.5) | 35.9 | 3 | 2.2 (0.2, 8.0) | 69.3 |
| Maternal hypertension (pre-existing and gestational) | 275 |  |  | 115 |  |  |
| No | 218 | 81.8 (72.8, 88.7) | 4.7 | 92 | 83.8 (71.0, 92.5) | 5.8 |
| Yes | 57 | 18.2 (11.3, 27.2) | 20.9 | 23 | 16.2 ( 7.5, 29.0) | 29.9 |
| Maternal diabetes (type I/II/gestational) | 276 |  |  | 113 |  |  |
| No | 235 | 86.9 (79.0, 92.7) | 3.7 | 101 | 87.6 (74.8, 95.4) | 5.2 |
| Yes | 41 | 13.1 (7.3, 21.0) | 24.8 | 12 | 12.4 (4.6, 25.2) | 36.5 |
| Maternal depression | 276 |  |  | 115 |  |  |
| Never | 120 | 40.9 (31.2, 51.3) | 12.0 | 65 | 54.1 (38.2, 69.3) | 13.4 |
| Ever | 156 | 59.1 (48.7, 68.8) | 8.3 | 50 | 45.9 (30.7, 61.8) | 15.7 |

^a^Unweighted sample size; ^b^Weighted prevalence and 95% confidence interval. ^c^Relative standard error of weighted prevalence with higher value indicates statistical unreliable estimation. Statistical analysis by a Rao-Scott Chi-square test.

**Supplemental Figure Legends**

**Figure S1.** Participant flow diagram with numbers presented as an unweighted sample size, PRAMS, 2016-2020.

**Figure S2.** Participant demographic, presented as a weighted prevalence and a corresponding 95% confidence interval, of CC-exclusive users, EC initiators, and quitters, PRAMS, 2016-2020.

**Figure S3.** Smoking status patterns among pregnant smokers who used CC exclusively prior to pregnancy: Proportion remaining CC-exclusive users vs transitioning to be EC initiators or quitters during pregnancy by amount of CC use 3 months prior to pregnancy.

**Figure S4.** Changes in the daily average amount of CC usage during pregnancy and after delivery from before pregnancy use, presented as a weighted prevalence and a corresponding 95% confidence interval, of CC-exclusive users, EC initiators, and quitters, PRAMS, 2016-2020.

**Figure S5.** Changes in the daily average amount of CC usage during pregnancy and after delivery from before pregnancy use, presented as a weighted prevalence and a corresponding 95% confidence interval, of EC initiators who became dual users or EC replacers, PRAMS, 2016-2020.

**Figure S6.** Participant health behavior characteristics, presented as a weighted prevalence and a corresponding 95% confidence interval, of CC-exclusive users, EC initiators, and quitters, PRAMS, 2016-2020.

**Figure S7.** Participant health conditions, presented as a weighted prevalence and a corresponding 95% confidence interval, of CC-exclusive users, EC initiators, and quitters, PRAMS, 2016-2020.

**Figure S8.** Participant neonatal outcomes, presented as a weighted prevalence and a corresponding 95% confidence interval, of CC-exclusive users, EC initiators, and quitters, PRAMS, 2016-2020.

**Figure S9.** Participant perinatal outcomes, presented as a weighted prevalence and a corresponding 95% confidence interval, of CC-exclusive users, EC initiators, and quitters, PRAMS, 2016-2020.

**Figure S10.** Neonatal outcomes, presented as a weighted prevalence and a corresponding 95% confidence interval, of EC initiators who became dual users or EC replacers, PRAMS, 2016-2020.
